# Supplementary material for: Assessing the impact of human trampling on vegetation: a systematic review and meta-analysis of experimental evidence
Source: PeerJ. 2014 May 1;2:e360. doi: 10.7717/peerj.360 (PMC4017817; doi:10.7717/peerj.360)
Supplement: Supplemental Information 1 [file peerj-02-360-s001.docx]

**Supplemental material 1.**

The calculation of Relative Vegetation Cover (RVC).

$$\frac{\begin{aligned} surviving cover on trampled subplots\times cf\times\\ 100\% \end{aligned}}{initial cover on trampled subplots}$$

where:

cf = $\frac{initial cover on control subplots}{surviving cover on control subplots}$
