# Supplementary material for: Assessing the impact of human trampling on vegetation: a systematic review and meta-analysis of experimental evidence
Source: PeerJ. 2014 May 1;2:e360. doi: 10.7717/peerj.360 (PMC4017817; doi:10.7717/peerj.360)
Supplement: Supplemental Information 3 — A methodological overview of articles not included in the meta-analysis, but which used a comparator or control in their experimental design. [file peerj-02-360-s003.docx]

# Supplemental material 3.

Overview of other studies containing comparators or controls, but not used in the meta-analysis. RCT = Randomised Controlled Trial; CT = Controlled Trial; SCS = Site Comparison Study; s.e. = standard error.

| **Reference** | **Study design** | **Habitat(s)** | **Baseline comparison** | **Intra treatment heterogeneity** | **Notes** | **Outcome(s) catalogued** | **Replicates** |
| --- | --- | --- | --- | --- | --- | --- | --- |
| Bayfield (1979) | RCT | Montane heath (Scotland) | No heterogeneity reported. | None reported. | Summer & winter treatments. No s.e. presented. | Vegetation cover; bare ground. | 3 |
| Bowles & Maun (1982) | RCT | Heath-grassland and sand dunes (Canada) | Homogenous vegetation reported. | None reported. | Trampling at different frequencies investigated. | Point-sampling of vegetation cover. | 4 |
| Liddle & Thyer (1986) | RCT | Sclerophyll forest (Australia) | Relatively homogenous field-layer reported. | None reported. | No true replication of treatments. Burning used as co-treatment. | Vegetation cover; species richness; soil characteristics. | 1 |
| Cole (1987) | RCT | Montane forest and grassland (USA) | No heterogeneity reported. | None reported. | Trampling at different frequencies investigated. | Vegetation cover; bare ground; species richness; soil compaction. | 1/2 (depending on community) |
| Ikeda & Okutomi (1992) | RCT | Experimental field system (Japan) | No heterogeneity reported. | None reported. | Vegetation trampled from seed. | RVC; soil characteristics. | 2/3 (depending on experimental system) |
| Cole (1995b) | RCT | Alpine, subalpine and montane grass and shrubland; temperate coniferous and deciduous forest (USA) | No heterogeneity reported. | None reported. | Futher analysis of data from Cole 1995a. | RVC. | 4 |
| Cole (1995c) | RCT | Subalpine meadow, and coniferous forest; low-elevation hard-wood forest (USA) | No heterogeneity reported. | None reported. | Data not presented. | RVC; Relative Vegetation Height. | 4 |
| Whinam & Chilcott (1999) | RCT | Alpine shrubland, grassland, bolster heath and fen (Tasmania) | No heterogeneity reported. | None reported. | S.e. not reported. | Biomass of broken plant material; vegetation cover; change in surface profile; track monitoring. | 1 |
| Hartley (2000) | RCT | Subalpine meadow (USA) | No heterogeneity reported. | None reported. | No s.e. reported. Monitoring over 30 years. | Vegetation cover; litter cover; bare ground. | 4 |
| Kutiel et al. (2000) | RCT | Coastal woodland (Israel) | No heterogeneity reported. | None reported. | No s.e. reported. | Vegetation cover (total and per species); vegetation height; species richness; soil characteristics. | 4 |
| Gallet & Rozé (2001) | RCT | Heathland (France) | Plant community composition homogenous. | Trampling on one day. | Winter and summer trampling performed. | RVC. | 3 |
| Thurston & Reader (2001) | RCT | Deciduous forest (Canada) | Microtopography homogenous. | None reported. | Biking and hiking treatment data combined. | Vegetation cover; bare ground; species richness. | 1 |
| Gallet & Rozé (2002) | RCT | Heathland (France) | Plant community composition homogenous | Trampling on one day; location homogenous | Deals with recovery from Gallet & Rozé (2001). | RVC. | 3 |
| Talbot et al. (2003) | RCT | Tropical rainforest (Australia) | No heterogeneity reported. | None reported. | Experiment conducted during dry season. | Vegetation cover; litter cover; soil characteristics. | 4 |
| Ros et al. (2004) | RCT | Pine woodland (Spain) | Vegetation cover and community composition all similar. | Location on slope varied. | No s.e. reported. | Vegetation cover; soil characteristics. | 3 |
| Bell & Bliss (1973) | CT | Montane and alpine (USA) | No heterogeneity reported. | Community type, location and altitude all homogenous. | None. | Productivity. | 2/3 (depending on community) |
| Harrison (1981) | CT | Semi-natural grassland and heathland (England) | Vegetation cover and plant community composition both homogenous. | Vegetation cover, location, community type both homogenous. | Winter and summer trampling performed. | RVC. | 1/2 (depending on community) |
| Kobayashi et al*.*(1997) | CT | Forest (Japan) | Vegetation cover homogenous. | None reported. | Different shade conditions at different sites. | Vegetation and soil measurements. | 12 |
| Arnesen (1999) | CT | Grassland and heathland (Norway) | Vegetation cover, community composition and soil homogenous. | Trampling treatments partly estimated, location homogenous. | 2 sites lacked controls. | Flowering shoots, above- and below-ground biomass. | 1 |
| Whinam & Chilcott (2003) | CT | Alpine and sub-alpine (Australia) | Plant community composition homogenous. | Trampling on one day; location homogenous. | Some sites underwent re-trampling. | Vegetation cover and biomass measurements. | 1 |
| Dale & Weaver (1974) | SCS | Montane forest and meadow (USA) | Location homogenous. | Location homogenous. | Number of users estimated from Park records. | Width and depth of trails. | 23 |
| Crawford & Liddle (1977) | SCS | Riparian meadow (England) | Location homogenous. | Location homogenous. | Meadow sprayed with MCPA. | Vegetation and soil measurements. | 3 |
| Bayfield et al*.*(1981) | SCS | Montane and laboratory (Scotland) | No heterogeneity reported. | None reported. | Trampling intensities estimated. | Damage/breakage. | 1 |
| Pounder (1985) | SCS | Montane and alpine (Norway) | Location, vegetation homogenous. | Location, vegetation homogenous. | None. | Path characteristics, vegetation, soil erosion. | 5 |
| Aspinall & Pye (1987) | SCS | Limestone grassland (England) | Different locations compared. | Within location, vegetation homogenous. | None. | Species richness. | 2 |
| Jim (1987) | SCS | Woodland (China) | No heterogeneity reported. | None reported. | Trampling intensities estimated. | Vegetation and soil measurements. | 1 |
| Hall & Kuss (1989) | SCS | Deciduous forest (USA) | No heterogeneity reported. | None reported. | Trampling intensities estimated | RVC, and relative frequency of species. | 10 |
| Boucher et al.(1991) | SCS | Tropical rain forest (Costa Rica) | Location homogenous. | Location and vegetation homogenous. | Trampling intensities estimated. | Vegetation cover and diversity measures. | 4 |
| Parikesit et al. (1995) | SCS | Forested cliff edge (Canada) | Location and vegetation homogenous. | Location and vegetation homogenous. | Trampling intensities estimated. | Species richness and soil characteristics. | 6–15 depending on site |
| Scott & Kirkpatrick (1994) | SCS | Sub-Antarctic vegetation (Australia) | Location homogenous. | Location homogenous. | Trampling intensities estimated. | Species abundance, vegetation height, soil characteristics. | 50 |
| Klug et al*.*(2002) | SCS | Alpine pasture (Austria) | Location, soil, vegetation homogenous. | Location, soil, vegetation homogenous. | Trampling intensities estimated. | Vegetation structure and biomass. | At least 3 |
| Gremmen et al. (2003) | SCS | Sub-Antarctic vegetation (South Africa) | Location and vegetation homogenous. | Location and vegetation homogenous. | Trampling intensities estimated. | Vegetation and soil characteristics. | 50 |
| Andrés-Abellán et al. (2005) | SCS | Pine, oak and mixed woodland (Spain) | Location and vegetation homogenous. | Location and vegetation homogenous. | Trampling intensities estimated. | Vegetation and soil characteristics. | 2 minimum |
| Benninger-Truax et al*.* (1992) | SCS | Montane/subalpine forest (USA) | Location and vegetation homogenous. | Location and vegetation homogenous. | Trampling intensities estimated. | Species richness and vegetation cover. | 2 |
| Bhuju & Ohsawa (1998) | SCS | Urban forest (Japan) | Location homogenous. | Location homogenous. | Trampling intensities estimated. | Vegetation cover and height. | 1 |
| Dzwonko & Loster (1997) | SCS | Woodland vegetation (Poland) | Location homogenous. | Location homogenous. | Trampling intensities estimated. | Species richness and vegetation cover. | 105 |
| Gómez-Limón & de Lucio (1995) | SCS | Grassland (Spain) | Location homogenous. | Location homogenous. | Trampling intensities estimated and grazing present. | Vegetation cover. | 10 |
| Kitazawa & Ohsawa (2002) | SCS | Broad leaved forest (Japan) | Location homogenous. | Location homogenous. | Trampling intensities estimated. | Vegetation cover and height. | 7 |
| Lämsä & Fritze (2003) | SCS | Urban forest (Finland) | Location, soil, vegetation homogenous. | Location, soil, vegetation homogenous. | Trampling intensities estimated. | Species richness; vegetation and soil measurements. | 4 |
| Li et al*.* (2005) | SCS | Forest (China) | No heterogeneity reported. | None reported. | Trampling intensities estimated. | Trail widening, root exposure. | 1 |
| McDougall & Wright (2004) | SCS | Feldmark vegetation (Australia) | Location, soil, vegetation homogenous. | Location, soil, vegetation homogenous. | Trampling intensities estimated. | Species abundance. | 50 |
| Rodgers & Parker (2003) | SCS | Dune/forest (USA) | No heterogeneity reported. | None reported. | Disturbance intensities estimated. | Species richness/vegetation cover. | 6 |
| Roovers et al. (2004) | SCS | Heathland/grassland (Belgium) | Location and vegetation homogenous. | Location and vegetation homogenous. | Trampling intensities estimated. | Floristic dissimilarity and species diversity. | 10 |
| Waltert et al. (2002) | SCS | Forest (Switzerland) | Location, soil, vegetation homogenous. | Location, soil, vegetation homogenous. | Trampling intensities estimated. | Species composition and vegetation cover. | 4 |

**References**

Andrés-Abellán, M., del Alamo, J.B., Landete-Castillejos, T., López-Serrano, F.R., García-Morote, F.A., and del Cerro-Barja, A. (2005) Impacts of visitors on soil and vegetation of the recreational area "Nacimiento del Rio Mundo" (Castilla-La Mancha, Spain). *Environmental Monitoring and Assessment,* **101**, 55-67.

Arnesen, T. (1999) Vegetation dynamics following trampling in grassland and heathland in Solendet Nature Reserve, a boreal upland area in Central Norway. *Nordic Journal of Botany,* **19**, 47-69.

Aspinall, R.J. & Pye, A.M. (1987) The effect of trampling on limestone grassland in the Malham area of North Yorkshire. *Journal of Biogeography,* **14**, 105-115.

Bayfield, N.G. (1979) Recovery of four montane heath communities on Cairngorm, Scotland, from disturbance by trampling. *Biological Conservation,* **15**, 165-179.

Bayfield, N.G., Urquhart, U.H., & Cooper, S.M. (1981) Susceptibility of four species of *Cladonia* to disturbance by trampling in the Cairngorm Mountains, Scotland. *Journal of Applied Ecology* **18**, 303-310.

Bell, K.L. & Bliss, L.C. (1973) Alpine disturbance studies: Olympic national park, USA. *Biological Conservation,* **5**, 25-32.

Benninger-Truax, M., Vankat, J.L., & Schaefer, R.L. (1992) Trail corridors as habitat and conduits for movement of plant species in Rocky Mountain National Park, Colorado, USA. *Landscape Ecology,* **6**, 269-278.

Bhuju, D.R. & Ohsawa, M. (1998) Effects of nature trails on ground vegetation and understory colonization of a patchy remnant forest in an urban domain. *Biological Conservation,* **85**, 123-135.

Boucher, D.H., Aviles, J., Chepote, R., Domínquez Gil, O.E., & Vilchez, B. (1991) Recovery of Trailside Vegetation from Trampling in a Tropical Rain-Forest. *Environmental Management,* **15**, 257-262.

Cole, D.N. (1987) Effects of three seasons of experimental trampling on five montane forest communities and a grassland in western Montana, USA. *Biological Conservation,* **40**, 219-244.

Cole, D.N. (1995b) Experimental Trampling of Vegetation .2. Predictors of Resistance and Resilience. *Journal of Applied Ecology,* **32**, 215-224.

Cole, D.N. (1995c) Recreational trampling experiments: effects of trampler weight and shoe type. *US Department of Agriculture Forest Service,* Research Note INT-425.

Crawford, A.K. & Liddle, M.J. (1977) The effect of trampling on neutral grassland. *Biological Conservation,* **12**, 135-142.

Dale, D. & Weaver, T. (1974) Trampling effects on vegetation of the trail corridors of north Rocky Mountain forests. *Journal of Applied Ecology,* **11**, 767-772.

Dzwonko, Z. & Loster, S. (1997) Effects of dominant trees and anthropogenic disturbances on species richness and floristic composition of secondary communities in southern Poland. *Journal of Applied Ecology,* **34**, 861-870.

Gallet, S. & Roze, F. (2001) Resistance of Atlantic Heathlands to trampling in Brittany (France): influence of vegetation type, season and weather conditions. *Biological Conservation,* **97**, 189-198.

Gallet, S. & Roze, F. (2002) Long-term effects of trampling on Atlantic Heathland in Brittany (France): resilience and tolerance in relation to season and meteorological conditions. *Biological Conservation,* **103**, 267-275.

Gómez-Limón, F. J. & Delucio, J.V. (1995) Recreational Activities and Loss of Diversity in Grasslands in Alta-Manzanares-Natural-Park, Spain. *Biological Conservation,* **74**, 99-105.

Gremmen, N.J.M., Smith, V.R., & van Tongeren, O.F.R. (2003) Impact of Trampling on the Vegetation of Subantarctic Marion Island. *Arctic, Antarctic, and Alpine Research,* **35**, 442-446.

Hall, C.N. & Kuss, F.R. (1989) Vegetation alteration along trails in Shenandoah National Park, Virginia. *Biological Conservation,* **48**, 211-227.

Harrison, C. (1981) Recovery of lowland grassland and heathland in southern England from disturbance by seasonal trampling. *Biological Conservation,* **19**, 119-130.

Ikeda, H. & Okutomi, K. (1992) Effects of species interactions on community organization along a trampling gradient. *Journal of Vegetation Science,* **3**, 217-222.

Jim, C.Y. (1987) Trampling Impacts of Recreationists on Picnic Sites in a Hong-Kong Country Park. *Environmental Conservation,* **14**, 117-127.

Kitazawa, T. & Ohsawa, M. (2002) Patterns of species diversity in rural herbaceous communities under different management regimes, Chiba, central Japan. *Biological Conservation,* **104**, 239-249.

Klug, B., Scharfetter-Lehrl, G., & Scharfetter, E. (2002) Effects of trampling on vegetation above the timberline in the Eastern Alps, Austria. *Arctic, Antarctic, and Alpine Research,* **34**, 377-388.

Kobayashi, T., Hori, Y., & Nomoto, N. (1997) Effects of trampling and vegetation removal on species diversity and micro-environment under different shade conditions. *Journal of Vegetation Science,* **8**, 873-880.

Li, W., Ge, X., & Liu, C. (2005) Hiking trails and tourism impact assessment in protected area: Jiuzhaigou Biosphere Reserve, China. *Environmental Monitoring & Assessment,* **108**, 279-293.

Malmivaara-Lämsä, M. & Fritze, F. (2003) Effects of wear and above ground forest site type characteristics on the soil microbial community structure in an urban setting. *Plant and Soil,* **256**, 187-203.

McDougall, K.L. & Wright, G.T. (2004) The impact of trampling on feldmark vegetation in Kosciuszko National Park, Australia. *Australian Journal of Botany,* **52**, 315-320.

Parikesit, P., Larson, D.W., & Matthes-Sears, U. (1995) Impacts of trails on cliff-edge forest structure. *Canadian Journal of Botany,* **73**, 943-953.

Pounder, E.J. (1985) The effects of footpath development on vegetation at the Okstindan Research Station in Arctic Norway. *Biological Conservation,* **34**, 273-288.

Rodgers, J.C. & Parker, K.C. (2003) Distribution of alien plant species in relation to human disturbance on the Georgia Sea Islands. *Diversity and Distributions,* **9**, 385-398.

Roovers, P., Baeten, S., & Hermy, M. (2004) Plant species variation across path ecotones in a variety of common vegetation types. *Plant Ecology,* **170**, 107-119.

Scott, J.J. & Kirkpatrick, J.B. (1994) Effects of human trampling on the sub-Antarctic vegetation of Macquarie Island. *Polar Record,* **30**, 207-220.

Talbot, L.M., Turton, S.M., & Graham, A.W. (2003) Trampling resistance of tropical rainforest soils and vegetation in the wet tropics of north east Australia. *Journal of Environmental Management*, **69**, 63-69.

Thurston, E. & Reader, R.J. (2001) Impacts of experimentally applied mountain biking and hiking on vegetation and soil of a deciduous forest. *Environmental Management,* **27**, 397-409.

Waltert, B., Wiemken, V., Rusterholz, H., Boller, T., & Baur, B. (2002) Disturbance of forest by trampling: effects on mycorrhizal roots of seedlings and mature trees of Fagus sylvatica. *Plant and Soil,* **243**, 143-154.

Whinam, J. & Chilcott, N. (1999) Impacts of trampling on alpine environments in central Tasmania. *Journal of Environmental Management,* **57**, 205-220.

Whinam, J. & Chilcott, N.M. (2003) Impacts after four years of experimental trampling on alpine/sub-alpine environments in western Tasmania. *Journal of Environmental Management,* **67**, 339-351.
