# Supplementary material for: Assessing the impact of human trampling on vegetation: a systematic review and meta-analysis of experimental evidence
Source: PeerJ. 2014 May 1;2:e360. doi: 10.7717/peerj.360 (PMC4017817; doi:10.7717/peerj.360)
Supplement: Supplemental Information 4 — Lists of studies not using a comparator or control, and of those studies which could not be retrieved within the resource constraints of the project. [file peerj-02-360-s004.docx]

**Supplemental material 4.**

1. List of studies located by the systematic review without comparators or controls (time series analyses).

Bright, J.A. (1986) Hiker impact on herbaceous vegetation along trails in an evergreen woodland of Central Texas. *Biological Conservation,* **36**, 53-69.

Burden, R.F. & Randerson, P.F. (1972) Quantitative studies of the effects of human trampling on vegetation as an aid to the management of semi-natural areas. *Journal of Applied Ecology,* **9**, 439-457.

Florgard, C. (2000) Long-term changes in indigenous vegetation preserved in urban areas. *Landscape and Urban Planning,* **52**, 101-116.

Gibson, D. J., Adams, E.D. Ely, J.S., Gustafson, D.J., McEwan, D., & Evans, T.R. (2000) Eighteen years of herbaceous layer recovery of a recreation area in a mesic forest. *Journal of the Torrey Botanical Society,* **127**, 230-239.

Godefroid, S., Massant, W., Weyembergh, G., & Koedam, N. (2003) Impact of fencing on the recovery of the ground flora on heavily eroded slopes of a deciduous forest. *Environmental Management,* **32**, 62-76.

Hylgaard, T. (1980) Recovery of plant communities on coastal sand dunes disturbed by human trampling. *Biological Conservation*, **19**, 15-25.

Maschinski, J., Frye, R., & Rutman, S. (1997) Demography and population viability of an endangered plant species before and after protection from trampling. *Conservation Biology,* **11**, 990-999.

Roovers, P., Bossuyt, B., Gulinck, H., & Hermy, M. (2005) Vegetation recovery on closed paths in temperate deciduous forests. *Journal of Environmental Management,* **74**, 273-281.

Whinam, J. & Comfort, M. (1996) The impact of commercial horse riding on sub-alpine environments at Cradle Mountain, Tasmania, Australia. *Journal of Environmental Management,* **47**, 61-70.

Willard, B.E. & Marr., J.W. (1971) Recovery of alpine tundra under protection after damage by human activities in the rocky mountains of Colorado. *Biological Conservation,* **3**, 181-190.

1. List of studies which were located by the systematic review, but which were not retrievable within the resource constraints of the project.

Aguiar, F.C. & Ferreira, M.T. (2005) Human-disturbed landscapes: Effects on composition and integrity of riparian woody vegetation in the Tagus River basin, Portugal. *Environmental Conservation,* **32**, 30-41.

Azlin, Y.N. & Philip, E. (2004) Soil compaction and tree decline along a recreational forest trail in Malaysia. *Arboricultural Journal,* **27**, 239-243.

Blom, C. W. P. M. (1976). Effects of trampling and soil compaction on the occurrence of some Plantago species in coastal sand dunes. *Oecologia Plantarum*, **11**, 225-241.

Buchanan, K. (1976) Some effects of trampling on the flora and invertebrate fauna of sand dunes. Discussion papers in conservation: no.13, University College, London.

Chadee, D.R.M. (1988) The impact and management of access areas of protected and recreation landscapes in Britain: with particular reference to vegetation. Ph.D. Thesis, Imperial College, University of London.

Clapham, S.E. (1974) Recreational pressures on soils and vegetation on Loughrigg Fell, Ambleside. B.A. Thesis, University of Liverpool.

Cole, D.N. (1985) Recreational trampling effects on six habitat types in western Montana. *US Department of Agriculture Forest Service,* Research Paper INT-350.

Cole, D.N. (1988) Disturbance and recovery of trampled montane grassland and forests in Montana. *US Department of Agriculture Forest Service,*  Research Paper INT-389.

Cole, D.N. (1993) Trampling Effects on Mountain Vegetation in Washington, Colorado, New-Hampshire, and North-Carolina. *US Department of Agriculture Forest Service,* Research Paper INT-464.

Cole, D.N. & Trull, S.J. (1992) Quantifying vegetation response to recreational disturbance in the North Cascades, Washington. *Northwest Science,* **66**, 229-236.

Conkling, P.W., Leonard, R.E., & Schwartz, C. (1984). The response of plant species to low levels of trampling stress on the Islands of Maine. In: Conkling, P.W., Drury, W.H., & Leonard, R.E. (Eds.) *People and Islands: resource management issues for islands in the Gulf of* *Maine,* Maine, Island Institute.

Eckrich, C.E.. & Holmquist, J.G. (2000) Trampling effects on a seagrass assemblage: direct effects, response of associated fauna, and the role of substrate characteristics. *Marine Ecology Progress* *Series*, 201, 199-209.

Evans, G.E. (1988) Tolerance of selected bluegrass and fescue taxa to simulated human foot traffic. *Journal of Environmental Horticulture,* **6**, 10-14.

Goryshina, T.K. (1983) Effect of trampling during recreational loads on internal leaf and thallome structure in certain plants. *Soviet Journal of Ecology,* **14**, 192-198.

Greene, T.A. & Nichols, T.J. (1996) Effects of long-term military training traffic on forest vegetation in central Minnesota. *Northern Journal of Applied Forestry,* **13**, 157-163.

Hall, T.E. & Farrell, T.A. (2001) Fuelwood depletion at wilderness campsites: extent and potential ecological significance. *Environmental Conservation,* **28**, 241-247.

Holmes, D.O. (1978) Experiments on the effects of human urine and trampling on subalpine plants. *Recreational Impact on Wildlands: Conference Proceedings,* **1979**, 79-88.

Jewell, R.A. (1984) Plant growth responses to trampling on neutral grassland. Ph.D. Thesis, University of London.

Jones, M.L. (1994) The effects of atmospheric nitrogen, desiccation, competition and trampling on Racomitrium lanuginosum (Hedw.) Brid. M.Sc. Thesis, Bangor University.

Jones, V. (1985) Physiological response of turf grasses to trampling pressure. Ph.D. Thesis, University of Keele.

Kellomaki, S. & Saastamoinen, V.L. (1975) Trampling tolerance of forest vegetation. *Acta Forestalia Fennica,***147**, 5-19.

Kirby, A. (1997) The ecological effects of low intensity trampling on ancient woodland flora. B.Sc. Thesis, University of Lancaster.

Kodrik, M. (2004) Human-induced environmental loading in the National Park Mala Fatra Mts indicated through fine roots of the main forest stand. *Ekologia Bratislava,* **23**, 408-413.

Koning, C.O. (2005) Vegetation patterns resulting from spatial and temporal variability in hydrology, soils, and trampling in an isolated basin marsh, New Hampshire, USA. *Wetlands,* **25**, 239-251.

Kozlowski, T.T. (1999) Soil compaction and growth of woody plants. *Scandinavian Journal of Forest Research,* **14**, 596-619.

Kuss, F.R. (1983) Hiking boot impacts on woodland trails (trampling). *Journal of Soil & Water Conservation,* **38**, 119-121.

Kuss, F.R. & Graefe, A.R. (1985) Effects of recreation trampling on natural area vegetation. *Journal of Leisure Research,* **17**, 165-183.

Kuss, F.R. & Hall, C.N. (1991) Ground Flora Trampling Studies - 5 Years after Closure. *Environmental Management,* **15**, 715-727.

Kutiel, P. & Zhevelev, Y. (2001) Recreational use impact on soil and vegetation at picnic sites in Aleppo pine forests on Mount Carmel, Israel. *Israel Journal of Plant Sciences,* **49**, 49-56.

Lei, S.A. (2004) Soil compaction from human trampling, biking, and off-road motor vehicle activity in a blackbrush (Coleogyne ramosissima) shrubland. *Western North American Naturalist,* **64**, 125-130.

Liddle, M.J. (1973) The effects of trampling and vehicles on natural vegetation. Ph.D. Thesis, University College of North Wales.

Orton, L. (2000) The ecological impact of trampling. B.Sc. Thesis, Imperial College, University of London.

Potito, A.P. & Beatty, S.W. (2005) Impacts of recreation trails on exotic and ruderal species distribution in grassland areas along the Colorado Front Range. *Environmental Management,* **36**, 230-236.

Pradhan, P. & Tripathi, R.S. (1983) Competition between Trifolium repens and Paspalum dilatatum as related to trampling. *Acta Oecologica: Oecologia Plantarum,* **4**, 345-353.

Scoggins, S. (1998) Vegetation response on coastal ledges to Seabird feeding and trampling activities: Isle of Rum, Inner Hebrides. B.Sc. Thesis, University of Southampton.

Scott, D., Bayfield, N.G., Cernusca, A., & Elston, D.A. (2002) Use of a weighing lysimeter system to assess the effects of trampling on evapotranspiration of montane plant communities. *Canadian Journal of Botany,* **80**, 675-683.

Somsak, L., Kubicek, F., Haberova, I., & Majzlanova, E. (1979) The influence of tourism upon the vegetation of the High Tatras. *Biologia,* **34**, 571-582.

Sothern, E., Randerson, P., Williams, A. and Dixon, J. (1985) Ecological Effects of Recreation at Merthyr Mawr Dunes, South Wales. In *Focus on*

*Nature Conservation No. 13. Sand Dunes and Their Management*, P. Doody, ed. Nature Conservancy Council, Peterborough, United Kingdom.

Sternberg, M., S. L. Yu, et al. (2004). Soil seed banks, habitat heterogeneity, and regeneration strategies in a Mediterranean coastal sand dune. *Israel Journal of Plant Sciences*, **52**, 213-221.

Sunohara, Y. & Ikeda, H. (2003) Effects of trampling and ethephon on leaf morphology in trampling-tolerant Plantago asiatica and Eleusine indica. *Weed Research,* **43**, 155-162.

Sunohara, Y., Ikeda, S., Murata, Y., Sukurai, N., & Noma, Y. (2002) Effects of trampling on morphology and ethylene production in asiatic plantain. *Weed Science,* **50**, 479-484.

Tonnesen, A.S. & Ebersole, J.J. (1997) Human trampling effects on regeneration and age structures of Pinus edulis and Juniperus monosperma. *Great Basin Naturalist*, **57**, 50-56.

Van Ham, L.A. & Revel, R.D. (2002) Right-of-way disturbances and revegetation in Alpine Tundra: An evaluation of natural revegetation on Plateau Mountain, Alberta. In: Goodrich-Mahoney, J.W., Mutrie, D.F., & Guild, C.A. (Eds.) *Seventh International Symposium on Environmental Concerns in Rights-of-Way-Management,* **7**, 135-146.

Weslawski J.M., Urban-Malinga B., Kotwicki L., Opalinski K., Szymelfenig M., Dutkowski M. (2000) Sandy coastlines - Are there conflicts between recreation and natural values? *Oceanological Studies*, 29, 5-18.

Whitecotton, R.C.A., David, M.B., Darmody, R.G., & Prince, D.L. (2000) Impact of foot traffic from military training on soil and vegetation properties. *Environmental Management,* **26**, 697-706.

Winning, S. A. (1994) A quantitative study of the effects of human trampling on woodland vegetation. M.Sc. Thesis, University College, University of London.

Witkowska-Zuk, L. & Andrzejewski, A. (2002) Vegetation of forest trails as a bioindicator of recreational anthropopressure on forest phytocoenoses. *Folia Forestalia Polonica,* **44**, 35-54.

Zubkova, T.A., Abrukova, V.V., Goncharova, I.F., Kolomiets, A.V., Komissarov, E.S., Ponomarenko, E.V., & Smirnova, E.V. (1982) Influence of recreation on a brown forest soil in the Carpathian reserve, severe loading, trampling. *Moscow University Soil Science Bulletin,* **37**, 56-58.
