# Supplementary material for: Assessing the impact of human trampling on vegetation: a systematic review and meta-analysis of experimental evidence
Source: PeerJ. 2014 May 1;2:e360. doi: 10.7717/peerj.360 (PMC4017817; doi:10.7717/peerj.360)
Supplement: Supplemental Information 6 [file peerj-02-360-s006.doc]

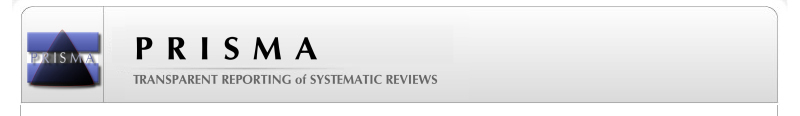
**PRISMA 2009 Flow Diagram**

**Screening**

**Included**

**Eligibility**

**Identification**

Records identified through database & bibliography searching
(n = 783 )

Additional records identified through other sources
(n = 0)

Records after duplicates removed
(n = 556)

Records screened
(n = 304)

Records excluded
(n = 252)

Full-text articles assessed for eligibility
(n = 145)

Full-text articles excluded with reasons (n = 43), or inaccessible (n = 48 (Supp Mat 4b))

Studies included in qualitative synthesis
(n = 54 (Supp Mat 3 & 4a))

Studies included in quantitative synthesis (meta-analysis)
(n = 9)
