# Supplementary material for: Assessing the impact of human trampling on vegetation: a systematic review and meta-analysis of experimental evidence
Source: PeerJ. 2014 May 1;2:e360. doi: 10.7717/peerj.360 (PMC4017817; doi:10.7717/peerj.360)
Supplement: Supplemental Information 2 — Trial identifiers, trampling intensities and Cohen’s d effect sizes (with 95% confidence intervals) are provided in this forest plot for closer inspection. [file peerj-02-360-s002.pdf]

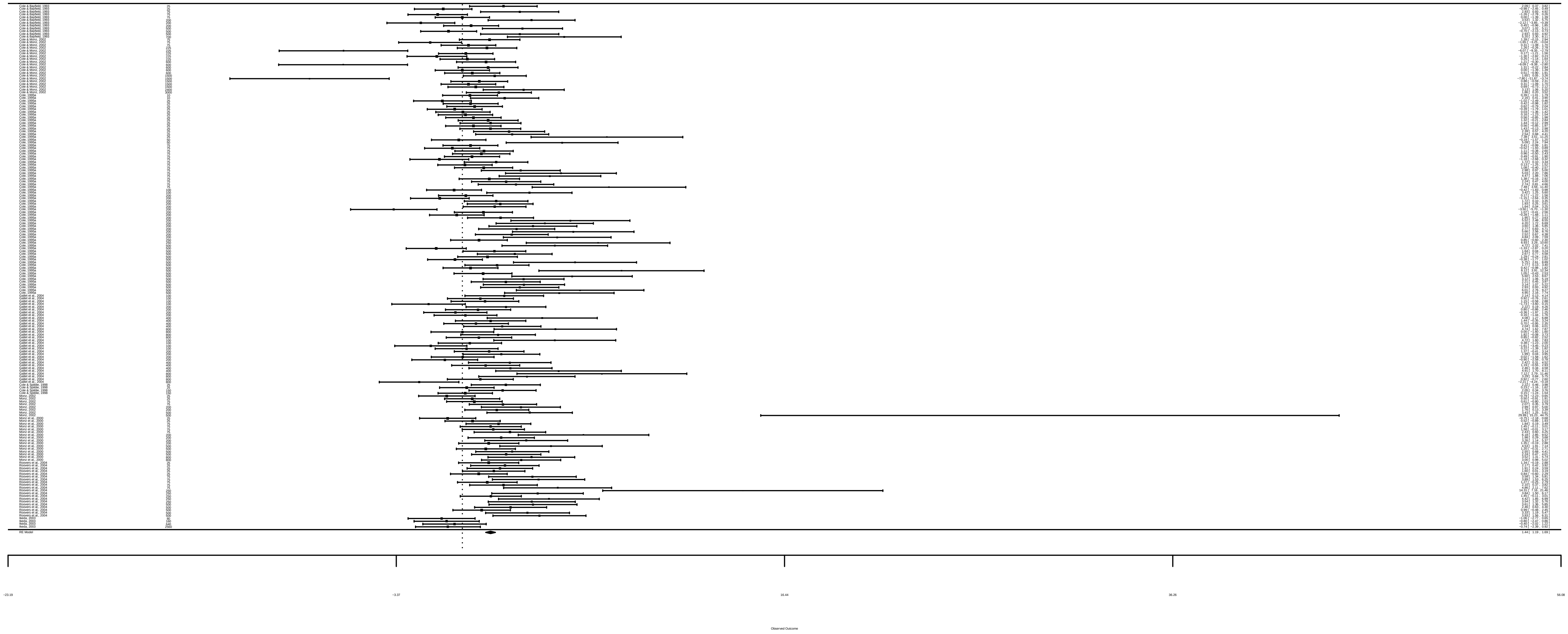

RE Model
−23.19
−3.37
16.44
36.26
56.08
Observed Outcome
